# Overview of Memristor-Based Design for Analog Applications

**DOI:** 10.3390/mi15040505

**Published:** 2024-04-07

**Authors:** Imen Barraj, Hassen Mestiri, Mohamed Masmoudi

**Affiliations:** 1 Department of Computer Engineering, College of Computer Engineering and Sciences, Prince Sattam Bin Abdulaziz University, Al-Kharj 11942, Saudi Arabia; 2 Systems Integration & Emerging Energies (SI2E), Electrical Engineering Department, National Engineers School of Sfax, University of Sfax, Sfax 3029, Tunisia; 3 Higher Institute of Computer Science and Multimedia of Gabes (ISIMG), University of Gabes, Gabes 6029, Tunisia; 4 Higher Institute of Applied Sciences and Technology of Sousse, University of Sousse, Sousse 4002, Tunisia; 5 Electronics and Micro-Electronics Laboratory, Faculty of Sciences of Monastir, University of Monastir, Monastir 5000, Tunisia

**Keywords:** memristors, analog design, programmable circuits, RF circuits

## Abstract

Memristor-based design has gained significant attention in recent years due to its potential to revolutionize various fields such as artificial intelligence, neuromorphic computing, non-volatile memory, signal processing, filtering, and radio frequency design. These emerging devices offer unique advantages such as non-volatile memory, low power consumption, and a high integration density. Their scalability and compatibility with existing fabrication processes make them an attractive option for industry adoption, paving the way for faster and more efficient architecture design. Researchers are actively exploring ways to optimize memristor technology for practical applications to harness its full potential. This includes developing novel materials and structures as well as improving the reliability and performance of memristors in various applications. This paper provides a comprehensive overview of the current advancements in memristor technology and their potential impact on the design of future electronic systems, focusing on its applications in the analog domain. By exploring the latest research and development in this field, researchers can gain valuable insights into how analog memristors can be integrated into their designs to achieve enhanced performance and efficiency. The paper delves into the fundamental principles of memristor technology, exploring its unique characteristics and advantages over traditional electronic components. It discusses the potential impact of memristors and challenges in the analog field of electronics, and highlights the progress made in their integration with existing circuitry, enabling novel functionalities and improved performance. Furthermore, it highlights ongoing research efforts to improve the performance and reliability of memristors, as well as the potential limitations and challenges that need to be addressed for widespread adoption, including variability in performance and reliability.

## 1. Introduction

Memristors, short for memory resistors, are electronic devices that can remember the amount of charge that has previously flowed through them. Leon Chua first theorized memristors in 1971 as the fourth fundamental circuit element, alongside resistors, capacitors, and inductors [1]. Unlike other circuit elements, memristors exhibit a unique property called memristance, which allows them to change their resistance based on the history of the applied voltage or current. This property makes memristors ideal for applications in non-volatile memory, neural networks, and analog computing. Memristors can mimic the synaptic behavior of biological neurons since they can remember past electrical activity. This makes them a promising technology for the development of neuromorphic computing systems.

The concept of the memristor was further developed and experimentally demonstrated in 2008 by a team of researchers at Hewlett-Packard (HP) Laboratories [2]. The memristor device developed by HP laboratories consists of stochastic titanium dioxide (TiO_2_) and oxygen-deficient (TiO_2x_) layers sandwiched between two platinum electrodes. This device successfully demonstrated the existence of the memristor predicted by Chua, establishing a connection between flux (Φ) and charge (q). The memristor resistance can vary between a low-resistance state (LRS) and a high-resistance state (HRS). These two states can be achieved by applying different voltage levels across the memristor. The resistance state R_ON_ corresponds to the LRS, where a significant current can flow through the memristor, representing the logical level ‘0’. On the other hand, the resistance state R_OFF_ corresponds to the HRS, where the current flow is significantly reduced, representing the logical level ‘1’. This difference in current flow between the LRS and HRS enables the memristor to store and retain information. A memristor retains its resistance value even when the power supply is disconnected. The most recently attained resistance is automatically saved in the memristor’s internal state, allowing it to resume its previous resistance value when power is restored. This unique characteristic of a memristor makes it ideal for applications requiring non-volatile memory. Additionally, retaining resistance values allows for efficient and reliable data storage in various electronic devices. Williams and Chua categorize all resistive devices with memory as memristors, regardless of the material or physical operation mechanism [3,4,5,6]. This categorization allows for a broader understanding and classification of various electronic resistive devices that utilize different storage mechanisms, such as phase-change materials, magnetic materials, and even biological systems. It also highlights the significance of memory in these devices, regardless of their specific properties or operational characteristics.

**Figure 1:**
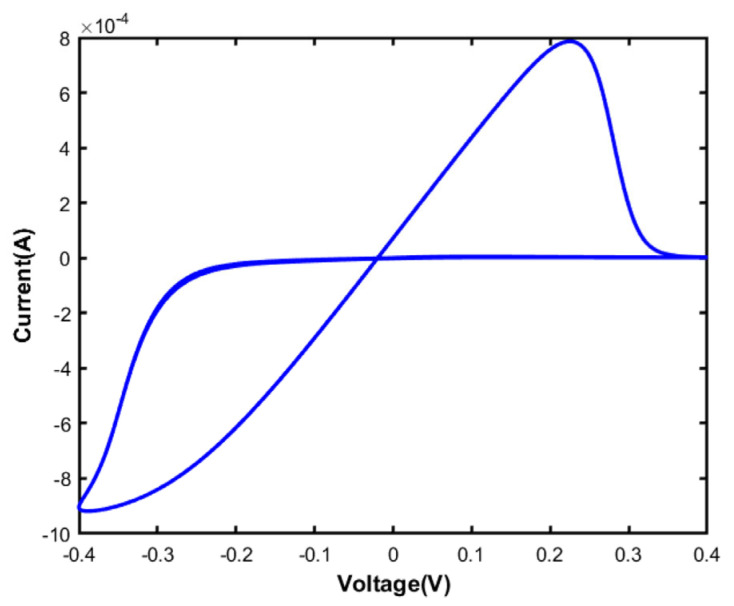
Memristor hysteresis loop [7].

A memristive device is characterized by a pinched hysteresis loop, which shows the relationship between the voltage applied across the device and the resulting current flowing through it, as shown in Figure 1 [7]. This unique hysteresis loop indicates that the memristive device has memory capabilities, allowing it to retain its resistance state even after the voltage is turned off. Also, the pinched hysteresis loop is a crucial feature distinguishing memristive devices from other electronic components. The shape of the hysteresis loop is altered by the frequency and amplitude of the periodic input signal applied to the memristive device. Higher frequencies and amplitudes can lead to a more distorted hysteresis loop, while lower frequencies and amplitudes result in a smoother loop. These variations can affect the performance and functionality of the memristive device in different applications. The memristor symbol consists of a rectangle with a diagonal line running from one corner to the opposite corner, representing the non-linear relationship between charge and flux.

Memristors have gained significant attention for a wide range of applications, as they can be used to create circuits that mimic the behavior of biological synapses. One prominent application is in the field of neuromorphic computing, where memristors can mimic the behavior of synapses in the human brain, enabling the development of efficient and powerful artificial neural networks [8,9,10,11,12,13]. These artificial neural networks can be used for tasks such as pattern recognition, image processing, and machine learning. Memristors have the potential to revolutionize the field of memory storage by providing faster and more energy-efficient alternatives to traditional storage devices like hard drives and flash memory. In addition, memristors have the potential to revolutionize artificial intelligence and machine learning by enabling the development of more efficient and powerful neural networks. Moreover, their non-volatile nature allows for instant-on capabilities and low power consumption, making them ideal for energy-efficient devices and Internet of Things (IoT) applications. Additionally, memristors are used for analog design and signal processing applications [13,14,15,16,17]. They offer the ability to store and manipulate continuous signals, making them suitable for applications such as audio and video processing, signal filtering, signal amplification, radio frequency design, and control systems. It allows for more accurate diagnoses and treatment planning. Furthermore, memristors have the advantage of being highly scalable and compatible with existing semiconductor fabrication processes, allowing for compact and efficient circuit designs. This makes them a promising technology for miniaturized electronic devices and integrated circuits. Also, their non-volatile nature ensures that data can be retained even when power is lost, making them suitable for applications requiring reliable and persistent storage. They are commonly used in various applications, such as telecommunications, mobile networks, medical imaging, healthcare monitoring, sensor data processing, and robotics. In the telecommunications industry and mobile networks, memristors are particularly utilized for storing and processing critical data efficiently and for firmware updates in network infrastructure equipment. Medical imaging and healthcare monitoring systems benefit from the compactness and reliability of these memory devices, which enable faster and more accurate image analysis and can help track vital signs and detect abnormalities in real-time. Additionally, memristors play a crucial role in sensor data processing by enabling quick and reliable data analysis for various applications. Lastly, memristors contribute to advancements in robotics by enhancing the performance and intelligence of robotic systems through efficient data storage and processing capabilities.

Various reviews on memristors have been published in the literature. These reviews cover topics such as memristor theory, history, properties, models, and fabrication techniques. Additionally, these reviews have highlighted the potential applications of memristors in the digital domain, including neuromorphic computing, artificial intelligence, logic operations, and memory storage devices. Recently, researchers’ efforts have focused on investigating new memristor-based analog design techniques and methodologies. By incorporating memristors into analog circuits, researchers hope to enhance the functionality and energy efficiency of various electronic systems, paving the way for innovative advancements in a wide range of fields. Additionally, these efforts aim to further expand the capabilities and applications of memristors beyond the digital domain. However, further research is needed to fully understand their behavior and optimize their performance for practical analog applications.

This review aims to provide a comprehensive overview of the current state of research on memristor-based analog circuits and highlight key challenges and opportunities for future development in this field. Memristors can revolutionize analog circuit design by enabling new functionalities and capabilities like adaptive filtering and reconfigurable circuits. These advancements could lead to significant improvements in communication systems, signal processing, and biomedical devices. The paper also discusses the challenges and prospects of memristor technology, emphasizing its potential for creating more efficient and powerful systems. Overall, the potential of memristors in analog applications is vast and continues to drive innovation in the field of electronics.

The rest of the paper is organized as follows: Section 2 presents the principles, models, materials and characteristics of memristors. Section 3 discusses the various applications of memristors in the analog field. Section 4 presents the current challenges and limitations of memristor technology. Finally, Section 5 concludes the paper.

## 2. Memristor: Models, Material, and Structure Design

### 2.1. Memristor Modeling

Memristor models are mathematical representations used to describe the behavior and characteristics of memristors. These models help in understanding how memristors store and retain information, as well as how they can be utilized in various applications. Some commonly used memristor models include the ideal memristor model, which assumes a linear relationship between charge and flux, and the nonlinear ion drift model, which considers the movement of ions within the memristor material. These models provide valuable insights into the electrical properties of memristors and enable researchers to design more efficient and reliable devices. Additionally, memristor models include the window function model, which considers the non-linear behavior of memristors near their switching thresholds; the adaptive memristor model, which incorporates the ability of memristors to adapt and change their resistance based on external stimuli; the diffusive memristor model, which considers the diffusion of charged species in the memristor material; the filamentary memristor model, which focuses on the formation and dissolution of conductive filaments; the spintronic memristor model, which incorporates the spin degree of freedom in addition to charge and flux; and the synaptic memristor model, which simulates the behavior of biological synapses. These models have significantly advanced our understanding of memristor devices and their potential applications. By studying different memristor models, researchers can gain insights into the underlying mechanisms of these devices and explore their potential for use in future technologies.

**Figure 2:**
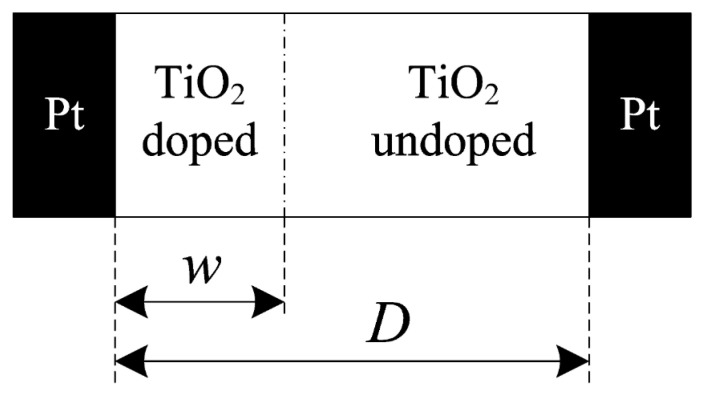
TiO_2_ memristor model [18].

The HP Labs-defined memristor physical model, which consists of a thin layer of TiO_2_ sandwiched between two metal electrodes, is depicted in Figure 2 [18]. This device exhibits resistance that can be increased or decreased depending on the polarity and magnitude of the applied voltage. In this experiment, one layer of TiO_2_ was doped with oxygen vacancies, while the other was left undoped. The presence of oxygen vacancies introduces defects, which can alter the conductivity of the memristor. This yields an insulator in the undoped region and a conductor in the doped region, creating a distinct boundary between the two layers. This boundary enables the formation of a conductive filament, which can be controlled by applying different voltages across the electrodes, allowing for precise control over memristor resistance programming. The quantity and direction of the electric charges passing through a memristor determine the width of the doped zone w(t) and the strength of the conductive filament. The width can range from zero to the memristor’s length (*D*). This control over the width of the doped zone enables the memristor to exhibit different resistance states, which allows for precise control over the flow of electric current through the device. By adjusting the width of the doped zone, the memristor can be switched between HRS and LRS, effectively controlling the flow of electrons. As a result, the memristor can be used as a programmable resistor in electronic circuits, offering potential applications in analog and digital fields. The total resistance of the memristor (*R_MEM_*) varies when the virtual barrier between the doped and undoped zones shifts [2]. This change in resistance is due to the movement of charged particles within the doped zone, which alters the conductivity of the memristor.

The expression for *R_MEM_* is given as follows:

(1) $$\;R_{MEM}\left(x\right)=R_{on}\left(x\right)+R_{off}(1-x)$$

where *x* = w/*D* ϵ[0, 1], *R_on_* is the memristor resistance when w = *D*, and *R_off_* is the memristor resistance when the value w is zero. The drift velocity is the speed of boundary movement between two nodes, expressed using the following state Equation [2]:

(2) $$\frac{dx}{dt}=k\;i\left(t\right)\;\;\;for\;k={µ}_{v}\frac{R_{on}}{D^{2}}$$

where ${µ}_{v}$ is the dopant mobility.

The studies reported in [6,8] demonstrated the non-linear behavior of implemented memristors. These studies showed that the resistance of memristors does not change linearly with applied voltage or current. Therefore, memristor models are mathematical representations used to describe the behavior and characteristics of memristors. These models help in understanding how memristors function and how they can be utilized in various applications. They consider factors such as voltage, current, resistance, and capacitance to accurately simulate the behavior of memristors in different scenarios. By using these models, researchers and engineers can predict and analyze the performance of memristors in complex circuits or systems. Additionally, these models can also aid in the design and optimization of memristor-based devices, enabling the development of more efficient and reliable technologies. The model proposed in [6] considers the non-linear relationship between the dopant drift and the resulting resistance change in memristors. Understanding the I–V characteristics can significantly enhance the design and optimization of memristor-based circuits and systems. The non-linearity of the drift velocity in [19,20,21,22] was simulated using the window function *f*(*w*), whose expression is given in Equation (3). This window function introduces a non-linear relationship between the applied voltage or current and the resulting resistance change in memristors, which aligns with the experimental findings. By incorporating this non-linearity into circuit design, engineers can better predict and control the behavior of memristor-based systems, leading to improved performance and efficiency.

(3) $$\frac{dw}{dt}=a\;f\left(w\right)\;V{\left(t\right)}^{m}$$

where *f*(*w*) is the window function, ‘*m*’ is an odd integer and ‘*a*’ is a constant.

In [8], the authors develop a more accurate physical model of the memristor, considering non-linear dopant drift and the voltage polarity’s impact on the device behavior. They also propose a modified HP model to capture dynamic memristor behavior under various operating conditions accurately. A resistor is coupled in series with a Simmons tunnel barrier instead of connecting two resistors in series [20]. This modification allows for a more realistic representation of the memristor’s behavior, particularly its resistance-switching dynamics. This model’s switching behavior is non-linear and asymmetrical, and its state equation is as follows:

(4) $$\frac{dx}{dt}=\left\{\begin{array}{c} C_{off}sinh\left(\frac{i}{i_{off}}\right)exp\left[-exp\left(\frac{x-a_{off}}{w_{c}}-\frac{\left|i\right|}{b}-\frac{x}{w_{c}}\right)\right]\\ C_{on}sinh\left(\frac{i}{i_{on}}\right)exp\left[-exp\left(\frac{x-a_{on}}{w_{c}}-\frac{\left|i\right|}{b}-\frac{x}{w_{c}}\right)\right] \end{array}\;\;\;\;\;\;\right\}$$

The state variable x represents the Simmons tunnel barrier width, and the fitting parameters are *C_off_*, *C_on_*, *a_off_*, *a_on_*, *w_c_*, and *b*. The memristor’s current thresholds are *i_off_* and *i_on_*.

The Simmons tunnel barrier model is more complicated, as shown in Equation (4), and as a consequence, it is computationally inefficient. This model introduces additional parameters and equations to describe the tunneling phenomenon accurately. However, its complexity requires more computational resources and time to solve compared to simpler models. In [21], the authors develop the ThrEshold Adaptive Memristor (TEAM) model to represent the same physical concept as the Simmons tunnel barrier model [19] in a more general and simpler manner. The TEAM model simplifies the description of the tunneling phenomenon by using a threshold adaptive approach, which reduces the computational complexity. This allows for more the efficient simulation and analysis of memristor behavior. A key advantage of the TEAM model is its ability to predict the memristor’s electrical characteristics accurately. The state equation of the TEAM model is represented by Equation (5) [21]:

(5) $$\frac{dx}{dt}=\left\{\begin{array}{c} K_{off}{\left(\frac{i\left(t\right)}{i_{off}}-1\right)}^{\alpha_{off}}\;\;\;f_{off}\left(x\right)\;,\;\;\;\;\;\;\;\;\;\;0<i_{off}<i\\ K_{on}{\left(\frac{i\left(t\right)}{i_{on}}-1\right)}^{\alpha_{on}}\;\;\;f_{on}\left(x\right)\;,\;\;\;\;\;\;\;\;\;\;i<i_{on}<0\\ 0\;,\;\;\;\;\;otherewise \end{array}\right\}$$

where *i_on_* and *i_off_* are current thresholds of the memristor, *K_off_*, *K_on_*, *α_off_* and αon are the fitting parameters, and *f_off_* (*x*) and *f_on_* (*x*) are the corresponding window functions of the memristor.

The experimental results from numerous memristive devices show voltage thresholds rather than current thresholds. These voltage thresholds indicate that changes primarily influence the switching behavior of memristive devices in the voltage applied across them. This finding suggests that controlling the voltage levels can effectively modulate the state of these devices. In [22], the TEAM model is extended to include the Voltage ThrEshold Adaptive Memristor (VTEAM) model. The VTEAM model allows for a more accurate representation of the behavior of memristor devices, as it incorporates voltage threshold adaptation. This extension provides a better understanding of how voltage changes can affect these devices’ state and functionality. Also, the VTEAM model is designed to mimic the behavior of biological synapses and enable synaptic plasticity in neuromorphic systems, allowing for dynamic adjustments of the voltage threshold, which enhances the device’s ability to learn and adapt to different input patterns. The VTEAM model is defined by Equation (6) [22]. The voltage dependency is identical to that in Equation (5), except that the voltage thresholds impact it.

(6) $$\frac{dx}{dt}=\left\{\begin{array}{c} K_{off}{\left(\frac{v\left(t\right)}{v}-1\right)}^{\alpha_{off}}\;\;\;f_{off}\left(x\right),\;\;\;\;\;\;\;\;\;\;0<v_{off}<v\\ K_{on}{\left(\frac{v\left(t\right)}{v_{on}}-1\right)}^{\alpha_{on}}\;\;\;f_{on}\left(x\right),\;\;\;\;\;\;\;\;\;\;v<v_{on}<0\\ 0,\;\;\;\;\;otherewise \end{array}\right\}$$

The VTEAM model is considered generic since it can be applied to any memristor model, regardless of its specific characteristics or behavior. This flexibility allows researchers and engineers to utilize the VTEAM model as a versatile tool for analyzing and simulating various memristor devices in different applications. Additionally, the VTEAM model’s adaptability enables it to accurately capture the dynamic behavior and non-linear properties of memristors, making it an invaluable resource in the field of memristor research. This flexibility allows for a comprehensive study of memristor devices and their performance across various operating conditions, making the VTEAM model valuable in memristor research.

Additionally, memristor modeling includes SPICE models, Verilog-A models, and behavioral models. These models allow researchers to simulate the behavior of memristors in different circuits and applications, providing valuable insights for design and optimization. Additionally, machine learning algorithms are being employed to further improve the accuracy and efficiency of memristor modeling. By incorporating machine learning techniques, researchers can enhance the predictive capabilities of these models and tailor them to specific applications. This intersection of memristor modeling and machine learning is paving the way for more sophisticated and reliable electronic devices in the future.

### 2.2. Memristor Material and Structure Design

Resistive switching (RS) materials are the most important part of memristors, and different RS materials have different resistive characteristics. It is crucial to understand the properties and behaviors of different RS materials in order to design and fabricate memristors with the desired performance. By studying the resistive characteristics of various materials, researchers can tailor memristors to specific applications and optimize their functionality. Recently, researchers have introduced new resistive switching (RS) materials for memristors. These include common electrodes and specific electrode materials, as well as organics, two-dimensional (2D) materials, three-dimensional (3D) integration, resistive layers, binary oxides, and perovskites. These new materials can be generally classified as inorganic and organic materials and offer improved performance in terms of speed, energy efficiency, and reliability, making them promising candidates for future electronic applications.

Memristor electrodes are materials used to carry electric current and participate in resistive reactions. They are made from various materials, including conventional metals, noble metals, alloys, carbon-based materials like graphene and carbon nanotubes, nitrides like TiN and TaN, and transparent conductive flexible oxides like indium tin oxide (ITO), doped ITO, and F-doped tin oxide (FTO) [23]. These electrodes can be classified into three types based on their role in resistive response (RS) behavior. First, electrodes conduct current without affecting RS behavior, using inert metals like Au and Pt. Second, electrodes create conductive filaments (CFs) through the chemical dissolution and deposition of active metal electrodes like Cu and Ag. Finally, certain new electrodes like ITO, FTO, graphene, TiN, and alloy electrodes aim to stabilize the RS behavior. Binary oxides, such as TiO_x_, SiO_x_, AlO_x_, NiO_x_, CuO_x_, ZnO_x_, HfO_x_, TaO_x_, WO_x_, ZrO_x_, and SnO_x_, are simple, highly stable, low in cost, and compatible with traditional CMOS processes [23]. HfO_x_ and TaO_x_ are promising due to their high on/off ratio, sub-ns operation speed, and endurance over 10^10^ cycles. However, conventional binary oxide memories often have high power consumption or low uniformity. In [24], a bismuth-doped tin oxide (Bi: SnO_2_) memristor is prepared using magnetron sputtering, resulting in smaller self-compliance currents, switching voltages, and operating currents compared to the ITO/SnO_2_/TiN device. However, conventional binary oxide memories often have high power consumption or low uniformity. The high-resistance state and low-resistance state have higher resistance values, and the operating current is reduced by over an order of magnitude. The authors of [25] created a three-layer memristor made of HfO_2_, BiFeO_3_(BFO), and HfO_2_ to improve memory, mimic how synapses work, and perform neuromorphic calculations. The memristor improved the storage window and multi-level storage capability, and the recognition simulation showed 91.2% accuracy. The BFO inserting layer facilitated the formation of hexagonal lattice structures of hafnium crystals. Research on binary oxides has been conducted by academics and industry, with various prototypes and applications being considered. Examples include Unity’s 64 Mb test chip, Panasonic’s MN101 L microcontroller (Osaka, Japan), Sony’s 16 Gb test chip (Tokyo, Japan), and TSMC’s embedded oxide memristor modules (Hsinchu, Taiwan) [23]. 

Perovskites are materials with an ABX_3_ structure, with a wide variety of physical and chemical properties achieved by partially doping the A and B sites with different valence or radius cations. Common perovskites include MAPbI_3_, (C_4_H_9_NH_3_)_2_PbBr_4_, LaFeO_3_, MAPbBr_3_, CH_3_NH_3_PbClXI_3_ [23], etc. These materials are ideal for solar cells, photodetectors, and light-emitting diodes due to their superior optoelectronic properties. They are also promising for designing next-generation neuromorphic memristors due to their mixed ion and electron conductivity, switchable majority carrier concentration, and slow photocurrent decay. However, perovskite materials lack environmental stability, which has been addressed by using lead-free double perovskite Cs_2_ AgBiBr_6_ for environmentally robust memristors (TO/Cs_2_AgBiBr_6_/Au structure) [26]. These memristors can last 10 s in alcohol burner flames and withstand temperatures of 180 °C or 60 °C γ-ray irradiation. Perovskite memristors, despite their extensive research, face limitations such as compatibility issues with CMOS processes, which restrict their practical applications.

The development of portable and wearable devices has led to the need for flexible memristors for data storage in electronic systems. Organic materials like organic small molecules, Ru-complexes, and polymers are promising candidates due to their low cost, solution processability, flexibility, and large-scale preparation. The authors of [27] prepared an Ag/2DP_BTA+PDA_/ITO memristor that exhibited good switching performance but had low endurance. The authors of [28] reported a new, flexible, and robust diffusion memristor based on a copolymer of trifluorochloroethylene and vinylidene fluoride (FK-800) Ag/FK-800/Pt, with an endurance of over 10^6^ cycles. However, their manufacturing yield remained low. In [29], a record yield of 90% for polymer memristors is achieved with miniaturization and low power consumption. The authors of [30] fabricated a solution-processed flexible organic memristor array with self-selectivity, achieving high recognition accuracy. Cha et al. prepared Cu/poly (1,3,5-trivinyl- 1,3,5-trimethylcyclotrisiloxane) (pV_3_D_3_)/Al_2_O_3_/Pt memristors, improving reliability.

Two-dimensional materials have gained attention due to their atomic-scale thickness, excellent electrical properties, and novel characteristics like flexibility and transparency. Researchers have investigated 2D memristors with vertical and planar structures, such as flexible memristors with vertical structures based on polyvinyl alcohol–graphene oxide hybrid nanocomposites [23]. These devices have great optical and electrical properties. They are also very stable under mechanical stress and can be separated from the bulk 2D crystal because of weak van der Waals forces between layers. Additionally, 2D material-based memristors are typically prepared on silicon wafers or polymer substrates, but these are not cheap or biodegradable. Cellulose paper is a good alternative due to its biodegradability, low cost, recyclability, light weight, and mechanical flexibility. The authors of [31] prepared a paper-based flexible memristor device using a nanohybrid material with silver and copper electrodes, achieving 500 cycles and a switching ratio of ~10^4^. However, 2D materials face challenges such as a large area of preparation, instability in the ambient atmosphere, controllable doping, and the direct deposition of metal electrodes, which can lead to defects and large contact resistance.

Researchers are continuing to explore and optimize these materials to further enhance the capabilities of memristors in various electronic devices. One area of focus is improving the stability and reliability of memristors, particularly in high-speed applications. Additionally, efforts are being made to reduce the energy consumption of these devices to make them more sustainable for future technology. Furthermore, researchers are also investigating ways to scale down the size of memristors for use in smaller electronic devices. This miniaturization could lead to more efficient and powerful electronics in the future.

## 3. Memristive Analog Design

Memristor-based analog circuits have gained attention in recent years due to their potential for low-power and high-density integration. These circuits offer advantages over traditional digital potentiometers, such as improved linearity and faster response times. High-voltage pulses are used to control the memristor resistance and can be easily adjusted to achieve precise analog signal manipulation. Their ability to store information in resistance states makes them suitable for applications in various functions, such as amplification, filtering, and signal processing. Also, they offer advantages such as low power consumption, high speed, and a compact size compared to traditional analog circuits. Memristive analog circuits have the potential to achieve higher levels of integration and density, making them suitable for applications requiring complex circuitry in a limited space. Furthermore, they maintain their state even in the absence of power, enabling non-volatile memory storage. This feature makes them ideal for applications that require data persistence and quick access to stored information. Additionally, memristive analog design offers flexibility and adaptability in various electronic systems through easy reconfiguration and programming.

This section explores the analog memristive circuits, including programmable circuits, DACs, and RF circuits, among others, that have been reported in the literature. These circuits have demonstrated impressive performance and energy efficiency, positioning them as a compelling choice for a wide range of electronic applications. Furthermore, the potential impact of these circuits on the field of electronics is significant, attracting significant interest and research.

### 3.1. Programmable Circuits

The study in [32] showcases the use of a digital emulator to implement memristors in practical memristive programmable analog circuits. The memristor emulator allows the simulation of memristor behavior in programmable analog circuits, providing a cost-effective solution for testing and prototyping. The proposed circuits can be easily built using inexpensive components and program properties. The proposed memristor emulator consists of a digital potentiometer, an analog-to-digital converter (ADC), and a microcontroller. The microcontroller calculates the resistance of the digital potentiometer using a code, while the ADC provides voltage for code calculation. This enables a current-controlled memristive system. The proposed memristor-based digital potentiometer circuit operates in analog mode, with terminals connected to ground and field effect transistors (FETs). FETs program the memristor state using control voltages V_pp_ and V_pn_, allowing the resistance to vary in the direction of the applied voltage. FETs also enable memristor tuning when the programming voltage V_pr_ exceeds the memristor’s threshold voltage V_T_. By controlling V_pr_, the memristor can be programmed to store and retain different resistance states. Different memristive analog circuits have been implemented to evaluate the performance and capabilities of these devices, including a programmable threshold comparator, a programmable gain amplifier, a programmable switching threshold Schmit trigger, and a frequency relaxation oscillator. The performance of these circuits using memristors has shown improved efficiency, lower power consumption, and enhanced functionality compared to circuits using conventional components.

**Figure 3:**
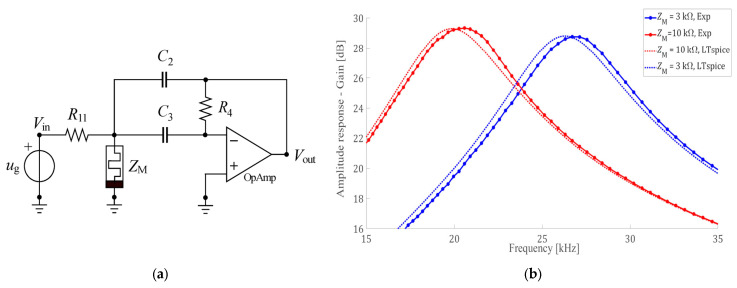
Tunable active memristor-based bandpass filter (**a**) schematic, and (**b**) measured amplitude response [33].

The authors in [33] present a solution for the automated programming of a commercially available memristor in a tunable active bandpass filter design. They demonstrate the effectiveness of their approach through simulations and experimental results, proving the feasibility of using memristors in practical electronic applications. Additionally, the central frequency can be programmed during filter operation, eliminating the need for memristor state-switching during operation and allowing for real-time adjustments to the filter characteristics. This flexibility opens new possibilities for the design of adaptive filtering systems. Figure 3a [33] displays the proposed design. It includes a memristor-based tunable active bandpass filter that removes the memristor-based programming signal from the output of the circuit. To achieve bandpass characteristics, the design incorporates a combination of passive and active components, allowing for efficient filtering while maintaining signal integrity within the desired bandwidth. The passive components, namely RC filters, help to attenuate frequencies outside of the desired range, while the active components, namely operational amplifiers, provide gain and stability to the signal within the passband. The filter is tuned for central frequency adjustments, with two target central frequencies of 19.5 kHz (1st state) and 26 kHz (2nd state), and a 3 dB bandwidth of 3.18 kHz. The capacitors C2 and C3, as well as the resistors R11 and R4, maintain their respective values of 200 pF, 200 pF, 5 kΩ, and 500 kΩ for both target central frequencies. The memristor resistance (Z_m_) in the first state is 10 kΩ, while in the second state it is 3 kΩ. The simulated and measured amplitude responses of the filter for the two states are presented in Figure 3b [33]. As shown in Figure 3b [33], there is good agreement between the simulated and measured results, indicating that the filter design is accurate and reliable. This consistency validates the effectiveness of the filter in both states. The measured gains of 29.3 dB at 19.5 kHz and 28.8 dB at 26 kHz are lower than the simulated value of 29 dB using LT-SPICE, owing to parasitic shunt capacitances.

The authors in [34] discuss the design and analysis of a memristor-based high-pass filter/amplifier topology for analog signal processing applications. The memristor serves as the negative feedback element, and a high-pass R-C filter is the input impedance. This configuration allows for the precise control of the cutoff frequency and the amplification of high-frequency signals. By tuning the memristor resistance, the circuit gain varies from 0.5 dB to 14 dB, allowing for a wide range of amplification levels to be achieved. This flexibility makes the circuit suitable for a variety of applications where variable gain is required. Additionally, the constant cut-off frequency ensures consistent performance across different input signals, further enhancing its reliability and versatility in various circuit designs.

**Figure 4:**
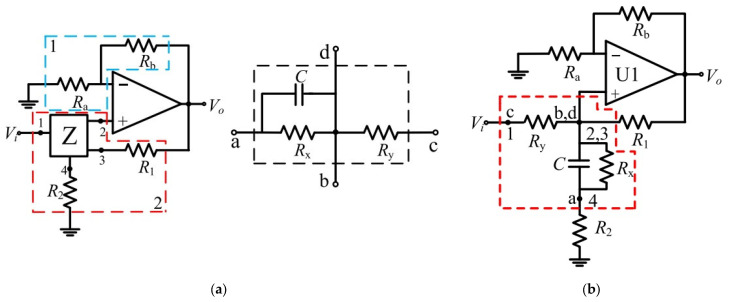
Proposed low-pass filter family: (**a**) Circuit and its Network Z, and (**b**) simulated topology A, and B-based circuit [35].

**Figure 5:**
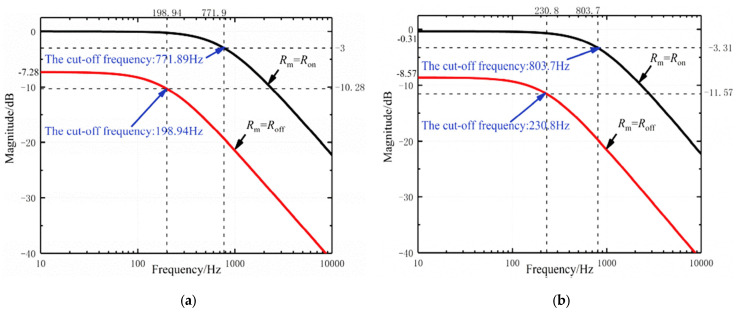
Experimental waveforms of magnitude–frequency characteristic of (**a**) topology A, and (**b**) topology B [35].

The authors in [35] propose a new method for hot-line adjusting cut-off frequencies using boundary values of memristance, integrating a preset DC voltage with the filtered signal to achieve the desired frequency adjustments. This approach eliminates the need for additional memristance write circuits, making it more suitable for integrated applications in various systems. A memristor emulator is implemented, allowing for the easy implementation and testing of the proposed approach. This emulator can accurately replicate the behavior of a memristor without the need for physical components, providing a cost-effective solution for researchers. Figure 4a [35] shows the proposed memrsitor low-pass filter design, which combines the connection nodes of a memristive circuit network Z with an op amp to achieve a low-pass filtering effect. The memristor network Z allows for programmable resistance values, providing flexibility in filter design and performance. The topology is divided into three parts: the feedback network, made up of two resistors *Ra* and *Rb*; the four-terminal network Z, with two resistors *R1* and *R2*; and the operational amplifier. The feedback network determines the gain of the amplifier. The inside circuit of network Z can be adjusted to create various filter configurations. Two configurations of the low-pass filter based on the circuit depicted in Figure 4b [35] are analyzed and tested to showcase the effectiveness of the suggested design. Theoretical analysis is conducted to determine the cut-off frequency and passband gain for each setting. The filter functionality is evaluated using square and sinusoidal voltage signals. The low-pass filter with positive feedback loop, named topology A, can be obtained by setting *R2* = 0 and replacing the resistor *Ry* with a memristor. Conversely, the low-pass filter without a positive feedback loop, named topology B, can be obtained by setting *R1* = ∞ and *R2* = 0. For experimental verification, the feedback resistances are adjusted to *Ra* = 5 kΩ and *Rb* = 1 kΩ, the capacitance is *C* = 1 µF, and the resistance is *Rx* = 1 kΩ. For topology A, the resistance *Rx* is set to 1 kΩ. Figure 5 [35] presents the experimental waveform magnitude–frequency characteristics of topologies A and B under the specified conditions. The memristor resistance varies between the boundary values Ron (lower memristance) and Roff (highest memristance). The filter’s cut-off frequency varies depending on the topology. Topology A has a lower cut-off frequency of 198.94 Hz with Roff and a higher cut-off frequency of 771.91 Hz with Ron. Topology B has a cut-off frequency of 230.8 Hz at Roff and 803.7 Hz at Ron. As demonstrated by the experimental results, the resistance values of the memristors affect the cut-off frequency of the filter. This flexibility in design allows for the customization and optimization of filter circuits for various purposes.

Table 1 summarizes the performances of the discussed memristor-based filters. Overall, the memristor-based filters showed promising results in terms of tunability and accuracy. Further research is needed to explore their potential applications in real-world systems. Additionally, more studies are required to investigate the long-term stability and reliability of these filters. Understanding their limitations and challenges will be crucial for successful integration into practical devices.

### 3.2. Memristive RF Circuits

New designs for wireless devices with multiple bands and standards require the reuse of RF chains to minimize their size and power consumption. Memristive components can be used in RF-programmable circuits in multi-band radios. These components maintain their resistance values even in the absence of power, rendering them ideal for RF-programmable circuits. In addition, memristive components offer the advantage of adjusting their resistance values in real time, allowing for the dynamic reconfiguration of RF circuits. This flexibility enables seamless adaptation to changing frequency bands and standards without needing physical modifications or additional components. The following are some examples of memristor-based RF circuits published in the literature. These RF circuits demonstrate the potential use of memristive components in various applications.

#### 3.2.1. High-Performance RF Switches

Radiofrequency switches (RFSs) are electronic devices that use radiofrequency signals to control the flow of current in a circuit [38,39]. These switches are commonly used in telecommunications and wireless communication systems to route signals quickly and efficiently between different components and accurately switch between different frequency bands. They offer fast switching speeds, low power consumption, and high reliability, making them ideal for applications that require rapid signal switching and minimal power loss. Furthermore, complex systems can seamlessly integrate RFSs with other electronic components. Switches based on microelectromechanical systems and phase-change materials are being explored as potential solutions for achieving higher frequencies and improved performance in RF switches [40,41]. However, these devices suffer from disadvantages such as large physical dimensions and high actuation voltages. Additionally, these switches often have limited durability and reliability due to the mechanical nature of their operation. Efforts are being made to overcome these challenges by exploring alternative materials and designs, such as nanoelectromechanical systems, graphene-based switches, and memristor-based switches, which offer the potential for smaller sizes, lower actuation voltages, and improved performance. These advanced switches also have the potential to enable new functionalities, such as reconfigurable antennas and cognitive radio systems. Furthermore, ongoing research focuses on developing RF switches capable of operating at higher frequencies, including millimeter-wave and terahertz frequencies. This would enable faster data transfer rates and support emerging technologies like 5G networks and wireless communication systems. Additionally, efforts are being made to improve the power handling capabilities of RF switches to meet the increasing demands of high-power applications in fields like radar systems and satellite communications.

Low ON resistance and minimal OFF capacitance are essential for low insertion loss and good isolation in RF memristive switches (RFMSs) [42]. Most RFMSs are electrochemical metallization memristors with asymmetric metal electrodes separated by a small gap or insulating layer [43]. This design enables precise control over the switching between high and low resistance states. Memristors are equivalent to parallel RC circuits, where R is the resistive state, and C is the between-electrode capacitance. Losses and substrate capacitance present higher-order effects [44]. These higher-order effects can affect the overall performance of RFMSs, leading to increased insertion loss and decreased isolation. Therefore, optimizing the design of RFMS to minimize these effects is crucial for achieving high-performance radio-frequency applications. Additionally, careful consideration of the materials used for the metal electrodes and insulating layer can further enhance the performance of RFMSs by reducing the parasitic capacitance and improving the switching speed.

In [45], a nanoscale RF switch is implemented using a nonvolatile memristive device with a pair of electrochemically asymmetric metal electrodes separated by a nanometer-scale air gap, which allows for the efficient and reliable switching of radio frequency signals. The use of a nonvolatile memristive device ensures that the switch retains its state even when the power is turned off, providing long-term stability and durability. Also, it allows for reliable and stable switching operations, while the asymmetric metal electrodes provide efficient control over the switch’s performance. The nanometer-scale air gap ensures minimal interference and maximizes the switch’s overall efficiency. The switch uses silver (Ag) as the active metal, offering low bulk resistivity and an effective dielectric constant, and its nanoscale feature size provides low insertion loss, high isolation, competitive linearity, and power-handling capabilities. The switch’s nanoscale feature size also allows for improved thermal management, reducing the risk of overheating and increasing its overall reliability. Furthermore, the use of silver as an active metal enhances the switch’s durability and longevity, making it suitable for long-term applications in various industries. The nanoscale RFMS can operate with a voltage as low as 0.4 V and boasts an ON/OFF conductance ratio of up to 10^12^. This nanoscale RFMS offers significant advantages in terms of low power consumption and high performance. Its ability to operate at such a low voltage makes it suitable for various applications, including energy-efficient electronics and sensor devices. The switch’s performance up to 110 GHz is measured, showing low insertion loss (0.3 dB at 40 GHz), high isolation (30 dB at 40 GHz), an average cutoff frequency of 35 THz, competitive linearity, and power-handling capabilities. Additionally, the RFMS exhibits excellent reliability and stability over extended periods of operation, making it suitable for a wide range of applications in high-frequency communication systems. Furthermore, the switch’s compact size and low power consumption make it an attractive choice for integration into portable electronic devices.

A non-volatile memristive RF switch with a Copper/Nafion/Gold structure on a coplanar waveguide is proposed in [46]. The design incorporates compensation for its parasitic effect and investigates the feasibility of its fabrication process. The compensation for parasitic effects ensures the reliable and efficient operation of the switch, further enhancing its performance. Full-wave simulations up to 10 GHz predict good performance, with ON-state insertion and return losses better than 0.54 and 25 dB, respectively, and OFF-state isolation higher than 22.8 dB. The proposed design also demonstrates low power consumption and high linearity, making it suitable for various applications in microwave and millimeter-wave systems. The simulation results validate the effectiveness of the compensation technique, confirming the feasibility of the fabrication process and highlighting the potential of this coplanar waveguide structure for practical implementation. In [47], a novel switching device for microwave systems using nanoionics is presented. The device uses an electrochemical process to form and remove a conductive metallic bridge, resulting in low insertion loss, isolation, voltage, power, and energy consumption. Its simple planar geometry allows for easy integration into microwave power distribution circuits.

Table 2 summarizes the performances of the discussed memristor-based RF switches. The table includes key metrics such as frequency range, insertion loss, and isolation for each switch design. These performance metrics provide valuable insights for comparing the efficiency and effectiveness of different memristor-based RF switches. Furthermore, the data presented in Table 2 allows for a comprehensive evaluation of the capabilities of each switch in terms of RF signal processing. Although substantial advances in the development of memristive RF switches have been made, several challenges must be addressed to advance the design of more efficient switches. One key challenge is improving the reliability and durability of memristor-based RF switches to ensure long-term performance. Additionally, enhancing the scalability and integration of these switches into complex RF systems is crucial for their widespread adoption in various applications. The endurance of memristive RF switches should be increased to ensure stable device functioning in practical communication and connectivity systems. Furthermore, optimizing the power consumption of memristive RF switches is essential to increasing efficiency and reducing energy usage. Overall, continued research and innovation in the field of memristive RF switch technology are necessary to overcome these challenges and unlock their full potential in modern communication systems.

#### 3.2.2. Tunable Inductors

RFMSs have demonstrated promising high-frequency performance, making them suitable for integrated circuit applications. The authors in [49] present two novel topologies of tunable inductors using MEmristors (TIME): a memristive-via-switched tunable inductor and a multi-layer stacked inductor switched by an RFMS single-pole double-throw. These topologies offer the advantage of tunability, allowing for adjustable inductance values to meet specific circuit requirements. The memristive-via-switched tunable inductor provides a compact solution, while the multi-layer stacked inductor offers increased inductance values without sacrificing performance. These passive inductor topologies are tuned by electrochemical metallization memristors, improving tunable inductors’ performance. As the memristor transitions from a low resistance state (LHS) to a high resistance state (HRS), the inductance changes from 2.43 nH to 9.6 nH, leading to a quality factor ranging from 12.4 to 18, respectively. The simulation results also show a maximum tunability of 296% and a self-resonant frequency above 8.4 GHz. These performance metrics make the memristive-via-switched tunable inductor and multi-layer stacked inductor highly desirable for applications requiring precise control over inductance values. Furthermore, the electrochemical metallization memristors enable the dynamic tuning of the inductors, allowing real-time adjustments to meet changing circuit requirements.

The authors in [50] explore the use of a memristor to control the biasing current of operational transconductance amplifiers (OTA) for gyrators. Gyrators are important components in electronic circuits that simulate inductors. By utilizing a memristor to control the biasing current of OTA, it is possible to enhance the performance and efficiency of gyrators. Additionally, this approach offers the advantage of flexibility and adaptability in adjusting the behavior of gyrators for various applications. The gyration constant is linearly controlled by the memristance state. This allows for precise control over the simulated inductance, making it easier to design circuits with specific frequency responses. Furthermore, the use of a memristor as a biasing current controller eliminates the need for external components, simplifies the circuit design, and reduces cost. The circuit is implemented using 0.18 μm CMOS technology from TSMC and simulated using Cadence Virtuoso. The circuit’s performance was evaluated through various simulations, including frequency response analysis and power consumption measurement. The off-chip memristor is a metal-oxide bi-layer with a non-volatile memristance range of 4.7 kΩ to 170 kΩ. The active inductance range is 95 μH to 1.55 mH, with an inductive bandwidth of 69 MHz and 18 MHz. The power consumption ranges from 0.21 mW to 1.95 mW, depending on the memristance and equivalent inductance.

Based on the discussed works, the memristor exhibits promising results in designing tunable inductors for radio frequency applications, but further research is needed to optimize their performance and reliability for practical communication systems. One potential area of improvement is reducing the variability in memristor behavior, which can impact the consistency of inductor performance. Additionally, exploring different materials and structures could enhance the memristor’s capabilities in terms of frequency range and power handling, allowing it to achieve higher efficiency and stability in RF applications. Furthermore, investigating novel fabrication techniques may also contribute to enhancing the overall performance of memristor-based inductors.

#### 3.2.3. CMOS Amplifier

**Figure 6:**
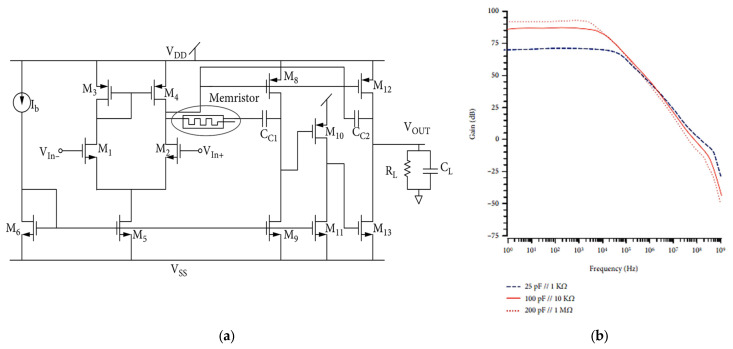
Proposed three-stage true-class AB amplifier: (**a**) schematic and (**b**) simulated gain [51].

The growing demand for multi-band and multi-standard wireless devices requires flexible designs that can support multiple frequency bands and communication protocols in a single device, such as Wi-Fi, Bluetooth, and cellular. In [51], a low-voltage high-gain wideband three-stage true-class AB amplifier is presented. The schematic of the proposed amplifier is depicted in Figure 6a [51] and designed using a 0.18 μm CMOS process. As shown in Figure 6b [51], the amplifier achieves a tunable high gain and operates at a low supply voltage of 1.8 V, making it suitable for low-power applications. The memristor-based pole-zero cancellation improves the amplifier’s performance in terms of linearity and distortion, while the nested Miller compensation technique helps to make it more stable and widen its bandwidth. The amplifier’s gain and phase margin are crucial for ensuring stability and performance across various load conditions. By analyzing the amplifier’s response to these different loads, engineers can optimize its design for maximum efficiency and reliability. The proposed amplifier operates with a ±1 V supply voltage and 175 μA bias current, which is capable of driving a wide range of loads and maintaining a phase margin of over 60°. Its unity gain frequency is 136.7 MHz, offering a high bandwidth without affecting the amplifier gain. Also, the simulation results show that the proposed amplifier is designed to handle a variety of load configurations to ensure optimal performance in different scenarios (200 pF//1 MΩ, 100 pF//10 KΩ, and 25 pF//1 KΩ), as depicted in Figure 6b [51]. The amplifier’s gain ranges from 70.5 dB to 92 dB. This flexibility allows for versatility in applications requiring varying impedance levels. Overall, this amplifier is designed to provide high performance and versatility in various applications. With its wide load-driving capability and excellent phase margin, it ensures stable and reliable operation even with demanding loads. Additionally, the high unity gain frequency allows for accurate signal reproduction and fast response times, making it suitable for applications requiring a high bandwidth.

A dual-band memristor-based Low-Noise Amplifier (LNA) is introduced in [52]. The proposed dual-band source-degenerated CS LNA implementation employs TIME inductors [49] as the gate and output inductors. TIME inductors allow for gain and frequency tunability, enhancing signal reception in the 2.4 GHz and 5 GHz WiFi bands. The LNA enhances signal reception in both frequency bands by providing high gain and low noise figures. The simulation results demonstrate that the LNA has a small area overhead and achieves high gains of 18.8 dB and 10.3 dB at 2.4 GHz and 5 GHz, respectively, while maintaining a low noise figure below 2.3 dB.

An optimized hybrid Operational Transconductance Amplifier (OTA) using source degeneration with adaptive biasing for high-frequency applications is presented in [53]. The proposed OTA design combines the benefits of source degeneration and adaptive biasing techniques to enhance its performance in high-frequency applications. The integration of these techniques results in improved linearity, reduced power consumption, a reduced die area, and enhanced stability, making it an ideal choice for demanding high-frequency circuits. The circuit operates with a supply voltage of ±1 V and a bias current. The linearity of the OTA is improved using memristor, which provides nonlinear behavior that can be adjusted to compensate for the nonlinearity inherent in the OTA itself. This allows for better accuracy and fidelity in high-frequency signal amplification, making it suitable for applications such as wireless communication or audio processing. Additionally, the use of a memristor also contributes to lower power consumption and the improved overall efficiency of the circuit. An OTA-C filter is designed and implemented, and a high-pass filter is demonstrated to showcase the effectiveness of the OTA in filtering out low-frequency signals. The use of OTA-C filters also allows for the easy adjustment of filter parameters, making them highly versatile for various signal processing applications.

A novel memristor-based compensation technique for an ultra-low-power single-output two-stage CMOS operational amplifier (Op-Amp) is presented in [54]. This technique utilizes the unique properties of memristors to enhance the performance of the Op-Amp, particularly in terms of power consumption and output stability. The Op-Amp uses a memristor programmer to regulate its compensation block’s memristance, improving the stability and phase margin. The simulation results show a 94 dB DC gain, a 1 MHz unity gain bandwidth, and 2.7 µW of consumption. The Op-Amp is designed in 0.18 μm CMOS technology, with a phase margin of 47 degrees and a unity gain bandwidth of 1 MHz. Furthermore, the proposed compensation technique effectively addresses the trade-off between performance and power consumption, making it suitable for low-power applications. The utilization of a memristor programmer in the regulation of the compensation block’s memristance not only enhances the stability and phase margin but also ensures efficient power utilization. These simulation results demonstrate the effectiveness of the Op-Amp design in achieving high performance with minimal power consumption in low-power applications.

Memristors offer a non-volatile memory element that can adjust resistance levels, providing more flexibility in amplifier performance. Reported studies have shown that incorporating memristors into CMOS amplifier designs can improve both gain and frequency range tunability. This advancement could potentially transform the field of analog circuit design, offering increased flexibility and efficiency over conventional techniques. However, the presented studies employ a CMOS memristor emulator or a memristor model created with Verilog_A or LTSpice to verify design performance, which may not accurately represent the behavior of real memristors. Therefore, further research is needed to validate the performance improvements of CMOS amplifiers with actual memristor components. Additionally, exploring different methods of integrating memristors into amplifier designs could lead to even more significant advancements in analog circuit design. 

### 3.3. Digital to Analog Converter

A data converter circuit using memristors and a DAC circuit for common source connected transistors is discussed in [55]. This circuit design combines the unique properties of memristors with the functionality of a DAC circuit. The memristors provide non-volatile memory and can be used to store and manipulate data, while the DAC circuit converts digital signals into analog voltages for driving common-source connected transistors. Analytical formulas are introduced to relate digital input to analog output, including transistor dimensions. These analytical formulas and PSpice simulations allow for the accurate prediction and optimization of the performance of the DAC circuit using memristors. This integration of memristors and DAC circuits opens new possibilities for efficient data storage and manipulation, as well as improved analog signal generation in various applications such as communication systems and audio devices. A modified 2-bit DAC circuit is introduced for overlapped inputs. The proposed design eliminates the need for complex signal processing algorithms, making it a cost-effective solution. Additionally, the integration of memristors into the DAC circuit enhances its scalability and potential for future advancements in data storage and analog signal processing technologies.

**Figure 7:**
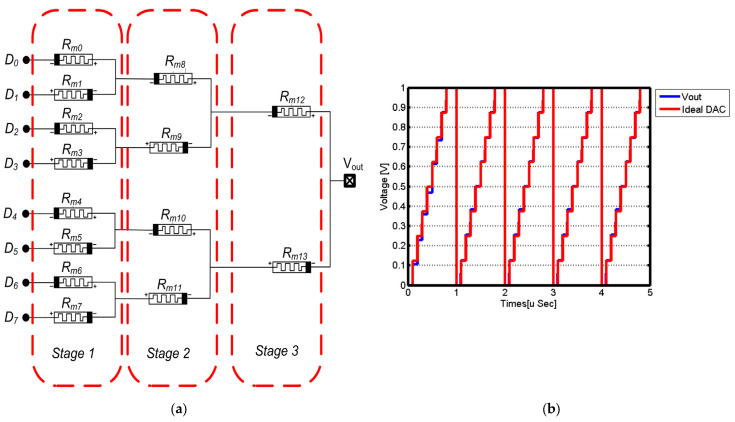
An eight-level input memristor-based DAC (**a**) circuit and (**b**) simulation [56].

**Figure 8:**
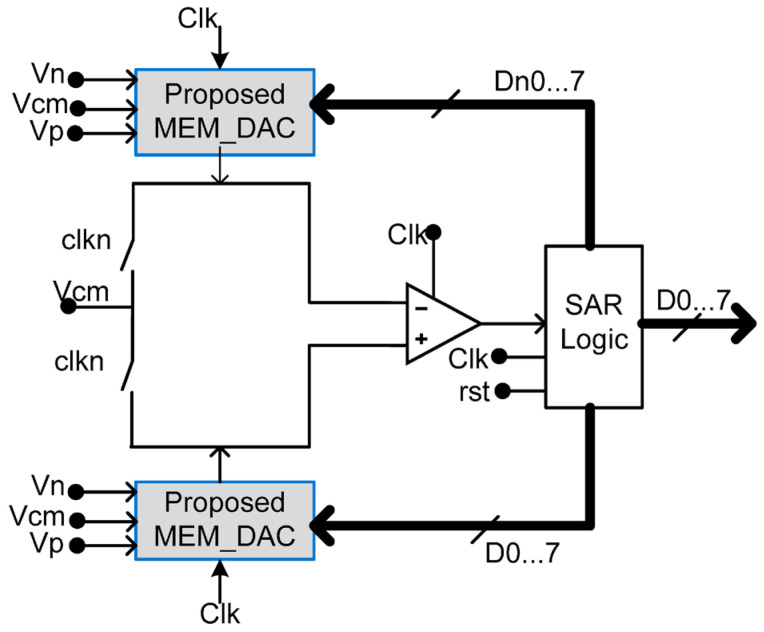
Proposed successive approximation register (SAR)–analog-to-digital converter (ADC) using memristor [56].

A memristor-based digital to analog converter (DAC) is proposed in [56] (Figure 7). The proposed memristive DAC offers improved performance and efficiency compared to traditional DACs. It utilizes the unique properties of memristors to achieve a higher resolution and lower power consumption. The proposed circuit utilizes two symmetric memristive DACs, enabling the design of both 4-bit and 8-bit DACs, as shown in Figure 8 [56]. Furthermore, the memristive DAC eliminates the need for complex calibration techniques, resulting in simplified circuitry and reduced manufacturing costs. The design reduces the area by 40% and uses an 8-bit memristive DAC in a successive approximation register analog-to-digital converter approach (SAR-ADC). The SAR-ADC achieves a signal-to-noise and distortion ratio of 49.3 dB and a spurious free dynamic range of 61 dB with a power supply of 1.2 V and a consumption of 21 µW. The figure of merit is 87.9 fj/Conv.-step. The designs were simulated with optimized parameters using the VTEAM model.

In [57], a memristor-based 4-bit digital-to-analog converter is presented. It uses a single input programmer with a deMultiplexer and reset switch to adjust the memristor’s resistance. The reset switch ensures the proper adjustment of the resistance, while the demultiplexer facilitates the selection of the desired memristor. This configuration enables precise control over the output voltage levels of the digital-to-analog converter. The converter achieves a maximum error of ±1% and achieves a DNL and INL of 0.075 LSB and 0.123 LSB, respectively. These low error values indicate the high accuracy and reliability of the digital-to-analog converter.

The authors in [58] present a design for a single-bit continuous-time Delta-Sigma modulator (Kennesaw, GA, USA) using a memristive finite impulse response (FIR) DAC in the feedback. The proposed design aims to improve the performance of Delta-Sigma modulators by leveraging the unique properties of memristive FIR DACs. By incorporating memristors in the feedback loop, the modulator can achieve higher linearity and lower power consumption compared to traditional designs. Additionally, the memristive FIR DAC offers greater flexibility in shaping the frequency response of the modulator, allowing for better noise shaping and improved overall performance. The 8-tap FIR filter coefficients are implemented using memristors with a programmable resistance ranging from 17.20 kΩ to 55.63 kΩ. This wide range of programmable resistance allows for the fine-tuning of the filter response, enabling precise control over the modulator’s performance characteristics. Furthermore, the use of memristors in the FIR DAC provides enhanced stability and reliability, as they have inherent non-volatile memory properties that can retain their resistance values even when power is removed. The modulator was designed and simulated using 180 nm standard CMOS technology and a memristor model. The simulation results show that the FIR DAC improves the modulator signal-to-noise and distortion ratio (SNDR) from 44.36 dB to 62.29 dB, even with 5 ns of RMS jitter at the sampling clock.

The presented studies highlight the potential of using memristors to implement digital-to-analog converters, yet they lack a comprehensive analysis of the possible challenges and limitations that could emerge during the implementation process. One issue with using memristors to design a digital-to-analog converter is their inherent variability, which can lead to inaccuracies in the conversion process. Additionally, the non-linear behavior of memristors can make it challenging to achieve consistent and reliable analog output. To address these challenges, researchers should explore techniques such as calibration and error correction algorithms to improve the accuracy and stability of memristor-based digital-to-analog converters. Additionally, advancements in material science and fabrication processes may help mitigate the variability and non-linearity issues associated with memristors. Despite these challenges, memristors still hold promise for advancing DAC technology due to their unique properties and potential for high-density integration.

### 3.4. Oscillators

A three-stage memristive PMOS ring oscillator is presented in [59,60]. The memristive PMOS inverter utilizes the unique properties of memristors to create a stable and reliable oscillator. The three-stage design ensures proper amplification and feedback to maintain the output pulses’ desired frequency and duty cycle. The memristive PMOS inverter is made up of a PMOS transistor in series with a memristor, which acts as a variable resistor to control the timing and amplitude of the output pulses. This combination allows precise control over the current flowing through the PMOS transistor, which determines the output waveform. Additionally, the memristor’s ability to retain its resistance value even when power is removed ensures the stability and reliability of the oscillator. The simulation results indicate that the RO output signal frequency varies from 3 to 5 GHz when the memristance decreases from 308.2 Ω to 55 Ω. The proposed memristive oscillator is used to generate ultra-wideband pulses. In this case, the oscillator can produce short-duration pulses with very high frequencies. The memristor is used to tune the frequency of the oscillator, allowing for flexibility in adjusting the output waveform. Furthermore, its simple design, low power consumption, compact size, and ease of implementation make it a cost-effective solution for high-frequency signal generation.

In [61], a low-power hybrid CMOS-Memristor Ring Oscillator (RO) with a higher randomness value is discussed. The researchers propose a novel design that combines CMOS technology with memristor devices to achieve low power consumption in the ring oscillator. The higher randomness value of the RO makes it suitable for applications such as secure communication and random number generation. A comparison between conventional CMOS inverters, memristive load inverters, and a two-memristive inverter-based RO in terms of the frequency of oscillation, power consumption, and randomness is given. The results show that the two-memristive inverter-based RO outperformed both the conventional CMOS inverters and memristive load inverters in terms of the frequency of oscillation. Additionally, it exhibited significantly lower power consumption compared to the other two designs. The higher randomness value of the two-memristive inverter-based RO also made it a promising candidate for applications requiring secure communication and random number generation. The proposed two memristor-based RO has a randomness of 51.16 μ, significantly lower than CMOS and memristive load-based designs. The maximum power of the design is reported at 270 μW. The lower power consumption of the proposed two memristor-based RO design makes it more energy efficient compared to CMOS and memristive load-based designs. Furthermore, the randomness value of 51.16 μ indicates a high level of unpredictability, making it suitable for cryptographic applications and enhancing security measures.

A memristor-based oscillator designed for potential analog applications is implemented in [62]. The circuit is designed to emulate the operation of a relaxation oscillator by passing a constant current in both directions through a memristor, which induces voltage variations due to resistance changes. These voltage fluctuations are subsequently fed back to the memristor, forming a feedback loop that enables the oscillation to continue. The ability of the memristor to store and recall resistance values allows for precise control over the oscillation frequency and waveform shape. The circuit is implemented using Winbond 90-nm CMOS and HfO2-ReRAM technology, with an operating frequency range of 300–1150 kHz, a power consumption of 700–830 μW, and a free-running oscillator jitter performance of 6.5 × 10^−5^. The proposed two-memristor inverter-based RO has a higher randomness value and improved power consumption. Additionally, the two-memristor inverter-based RO offers enhanced stability and reliability compared to traditional oscillators. This improvement in performance makes it suitable for various applications, such as communication systems, signal processing, and data encryption.

The discussed works investigate the use of memristors to design RF oscillator circuits for wireless communication applications. The studies focus on the advantages of using memristors in oscillator design, such as improved frequency tunability and reduced circuit complexity compared to traditional designs. The presented results are schematic simulations, demonstrating the feasibility and potential performance enhancements of memristor-based RF oscillators. Further experimental validation is needed to confirm the practicality of implementing these designs in real-world wireless communication systems. Several issues need to be addressed. One issue with using memristors to implement oscillators is their susceptibility to noise, which can affect the stability and performance of the oscillator. Another concern is the limited frequency range that memristors can operate at, which may restrict the potential applications of the oscillator in certain scenarios. Additionally, the non-linear behavior of memristors could introduce unwanted harmonics into the oscillator output, affecting signal quality. Furthermore, the variability in memristor characteristics can also lead to inconsistencies in frequency output, making it challenging to achieve precise and reliable oscillation. In order to mitigate these issues, careful design and characterization of memristors are necessary for successful implementation in RF oscillator circuits. Also, methods like adaptive tuning and feedback control can be used to fix the nonlinearities and variations in memristors, which makes the oscillator work better overall. Overall, addressing these challenges will be crucial for realizing the full potential of memristor-based RF oscillators in future electronic systems.

## 4. Challenges and Future Directions

Memristor technology has shown immense potential for revolutionizing analog and digital systems. However, there are still several limitations and challenges that need to be addressed for its widespread adoption. In this section, we will discuss some of the key obstacles facing memristor technology and potential solutions to overcome them. Additionally, we will explore current research efforts aimed at improving the performance and reliability of memristors in various applications.

Memristor-based analog circuits face reliability issues due to their susceptibility to variations in environmental conditions and manufacturing processes. These variations can lead to changes in the resistance levels of memristors, which affect the overall performance and accuracy of the analog circuits, making it challenging to ensure consistent and reliable operation in real-world applications. The difficulty in achieving consistent and reliable switching behavior can be attributed to two main factors: the non-ideal effects of analog resistive switching, which affect the overall performance, and the conductance drift or fluctuation of analog devices, which cause accuracy degradation over time. Thus, device reliability metrics can be categorized into basic and functional ones. Basic metrics focus on essential characteristics like retention, endurance, and random noise. Functional metrics, on the other hand, focus on the functional properties of analog design that influence circuit performance, like nonlinearity, asymmetry, dynamic range, precision, and variation. These metrics are crucial in evaluating the overall performance and quality of memristor-based analog devices, helping researchers identify areas for improvement and allowing for more informed decision making in the design and implementation of electronic systems. Notably, most published works focus on memristors’ use in implementing analog circuits without considering their reliability effect on design performance. Future research should aim to investigate the impact of memristor reliability on the long-term functionality and efficiency of analog devices, providing a more comprehensive understanding of their potential limitations. By addressing this gap in the current literature, researchers can develop strategies to enhance the reliability and robustness of analog memristor-based systems, ultimately leading to more reliable electronic devices in the future.

Additionally, there is a need for better understanding and control of device variability, as the performance of memristor devices can vary significantly from one device to another. This variability can impact the scalability and manufacturability of memristor technology, making it challenging to achieve consistent and reliable performance across a large-scale system. Furthermore, the integration of memristors into existing systems also poses challenges, as it requires significant modifications and adaptations to ensure the compatibility and optimal utilization of this emerging technology. Also, the variability in memristor devices can also affect their power consumption and overall energy efficiency, further complicating their integration into existing systems. Moreover, the complex fabrication processes involved in manufacturing memristors can lead to yield issues and higher production costs, which need to be addressed for the widespread adoption of this technology. Potential solutions and ongoing research efforts are focused on developing techniques to mitigate the variability in memristor technology. These include improving fabrication processes and materials, as well as implementing error correction algorithms to compensate for any inconsistencies. Additionally, researchers are exploring novel approaches to integrating memristors into existing architectures, such as hybrid systems that combine traditional computing elements with memristor-based components, to overcome compatibility challenges and enhance overall system performance. One approach being explored is the use of machine learning algorithms to optimize the performance and reliability of memristor-based systems. By training these algorithms on large datasets, researchers can identify patterns and trends that can be used to fine-tune the behavior of individual memristors and improve overall system stability.

Successful memristor-based design depends on reliable memristor–CMOS integration. In general, the operation of any memristor-based system requires CMOS circuitry to provide the necessary outputs. Thus, efficient memristor–CMOS integration is key to achieving any system gains. The integration process involves ensuring that the memristor and CMOS components work seamlessly together to maximize performance. Without proper integration, the system may not function optimally or may even fail to operate altogether. In the literature, different methods of integration have been described, such as chip-level integration, silicon vias, and the monolithic integration of memristor arrays directly on top of CMOS circuitry. Each approach has its own advantages and challenges, with researchers continuing to explore new methods for improving integration efficiency. One promising direction is the development of 3D integration techniques that stack memristor layers on top of CMOS layers to increase density and reduce the interconnect lengths. Also, advancements in materials science are enabling the creation of new types of memristors that can be more easily integrated with existing CMOS technology. Additionally, advancements in design automation tools are helping to streamline the integration process, allowing for the faster and more reliable implementation of memristor-CMOS systems.

Through decades of intensive research, memristors have been greatly developed. The economic feasibility and commercial viability of transitioning from traditional technologies to memristor-based technologies have been a topic of much debate in recent years. While memristors offer significant advantages such as lower power consumption and faster processing speeds, the initial investment required for implementation has been a major barrier for many companies. However, ongoing research and development efforts are aimed at reducing costs and improving performance, making the transition increasingly attractive for various industries. Collaborative efforts between researchers, engineers, and industry will be crucial in overcoming these challenges and unlocking the full potential of memristor technology. By working together, these partnerships can find innovative solutions to drive down costs and optimize performance, ultimately making memristors a more accessible and viable option for widespread adoption. In addition, the standardization and mass production of memristors will also play a key role in driving down costs and making them more accessible to a wider range of applications. As these advancements continue, the future looks promising for the widespread adoption of memristor technology in various sectors.

As discussed in this paper, there are various publications that demonstrate the theoretical advantages of memristor-based analog design, but more research is needed to provide empirical evidence of its effectiveness in real-world scenarios. Some works use memristor emulators or off-chip memristors to validate the theoretical advantages of the proposed approach design. However, these methods may not fully capture the complexities of integrating memristors into actual circuits. Therefore, further experimentation with on-chip memristors in practical applications is necessary to validate their potential benefits. Future studies should focus on integrating memristors into actual analog circuits to evaluate their performance in practical applications. This will help bridge the gap between the theoretical advantages and the real-world effectiveness of memristor-based analog design.

The long-term durability and stability of memristors are still areas of concern, as their performance may degrade over time. Researchers are also exploring novel materials and device architectures to address these challenges and improve the functionality of memristors. Efforts are being made to develop standardized testing methods and quality control measures to ensure consistent performance in commercial applications.

Another avenue of research involves developing new circuit designs that leverage the unique properties of memristors, such as their ability to store information even when the power is turned off. This opens possibilities for creating low-power, non-volatile memory systems that can retain data without the need for a constant power supply. Additionally, exploring the potential use of memristors in neuromorphic computing is an exciting area of research, as their ability to mimic synaptic behavior could lead to the development of highly efficient and powerful artificial intelligence systems.

## 5. Conclusions

This paper discusses the fundamental principles of memristor technology and its potential in analog applications, offering advantages such as improved energy efficiency and compactness. It explores the concept of memristance, which is the ability of a device to remember its previous resistance states, and how this unique property can revolutionize circuit design. By utilizing memristor technology, analog applications can benefit from enhanced performance and functionality. This technology has the potential to greatly improve energy efficiency and reduce the size of circuits, making it a promising option for various industries. Also, the use of memristors opens possibilities for adaptive and reconfigurable circuits. This flexibility in resistance values can lead to more efficient and versatile circuit designs. Additionally, it highlights the challenges and prospects of incorporating memristors into existing electronic systems. In conclusion, the integration of memristors into existing electronic systems holds great potential for enhancing performance and enabling new functionalities. As research continues to explore the capabilities of memristors, we can expect to see significant advancements in various technological applications.

## Figures and Tables

**Table 1:** Comparison of memristor-based filter.

Ref.	Type	Frequency Range	Memristor Type	Results Type
[33]	Band-pass filter	19.5 KHz–26 KHz	Commercially memristor	Measurement results
[34]	High-Pass Filter	50 KHz	TiO_2_ memristor model	Simulation results
[35]	Low-pass filter with positive feedback loop	198.94 Hz–771.91 Hz	Memristor emulator	Measurement results
	Low-pass filter without a positive feedback loop	230.8 Hz–803.7 Hz		
[36]	High pass filter	60 Hz	Memristor emulator	Simulation results
[37]	Low/High-pass filter	398 kHz–5.3 MHz	Printed memristor	Measurement results

**Table 2:** Comparison of memristor-based RF switches.

Features	[44]	[45]	[46]	[47]	[48]
**Frequency Range (GHz)**	10–40	0–40	0–6	0–10	0–3
**OFF-State Isolation (dB)**	36	35	30	26	16
**On-State insertion Loss (dB)**	0.26	0.33	0.5	0.54	1.1
**Minimum Line Width (um)**	-	<1	<1	50	100
**Results type**	Simulation results	Simulation results	Measurement results	Simulation results	Measurement results

## Data Availability

Data are contained within the article.
